# SAUV—A Bio-Inspired Soft-Robotic Autonomous Underwater Vehicle

**DOI:** 10.3389/fnbot.2020.00008

**Published:** 2020-02-21

**Authors:** Fabian Plum, Susanna Labisch, Jan-Henning Dirks

**Affiliations:** ^1^Department of Biomimetics, Hochschule Bremen - City University of Applied Sciences, Bremen, Germany; ^2^Department of Bioengineering, Imperial College London, London, United Kingdom; ^3^Biomimetic-Innovation-Centre, Hochschule Bremen - City University of Applied Sciences, Bremen, Germany; ^4^Max-Planck-Institute for Intelligent Systems, Stuttgart, Germany

**Keywords:** exoskeleton, genetic algorithm, compliant structure, shock absorbability, cave diving

## Abstract

Autonomous and remotely operated underwater vehicles allow us to reach places which have previously been inaccessible and perform complex repair, exploration and analysis tasks. As their navigation is not infallible, they may cause severe damage to themselves and their often fragile surroundings, such as flooded caves, coral reefs, or even accompanying divers in case of a collision. In this study, we used a shallow neural network, consisting of interlinking PID controllers, and trained by a genetic algorithm, to control a biologically inspired AUV with a soft and compliant exoskeleton. Such a compliant structure is a versatile and passive solution which reduces the accelerations induced by collisions to 56% of the original mean value acting upon the system, thus, notably reducing the stress on its components and resulting reaction forces on its surroundings. The segmented structure of this spherical exoskeleton protects the encased system without limiting the use of cameras, sensors or manipulators.

## Introduction

In recent years the use of Autonomous Underwater Vehicles (AUVs) and Remotely Operated Vehicles (ROVs) has become increasingly popular in marine biology and underwater exploration. These vehicles enable humans to reach depths and areas which would otherwise be too dangerous for divers or simply inaccessible (Hudson et al., 2005). Yet especially when having to navigate in complex or confined spaces, such as flooded caves, shipwrecks or coral reefs, conventional ROVs still show significant practical limitations. Most ROVs are trivially underactuated and therefore incapable of precisely maneuvering under such circumstances in the first place. The need for a cable connecting to a control station further reduces an ROVs ability to freely move in confined spaces. AUVs are free of this restriction, however, due to their limited on-board signal processing and navigation, they are still often unfit for the interaction with fragile surroundings or are themselves prone to take damage from unintentional collisions (Hudson et al., 2005; Hernández-Alvarado et al., 2016). Especially in fragile confined spaces such as underwater caves, a rigid object colliding with the environment can cause the release of debris or even rocks.

So far, these limitations were addressed using computationally, expensive technical solutions. Typical implementations include adding a multitude of sensors in order to capture the state of the system more accurately when planning its trajectory and additionally carrying out local behavior based strategies (Warren, 1990; Estes et al., 1996; Valavanis et al., 1997; Chyba et al., 2009). However, any active technical systems can fail, and, in these cases, a passive structure or mechanism should prevent severe consequences to the system and especially its environment. As a result, there is an increasing need for a fail-safe, computationally and economically inexpensive solution to allow safe maneuverability of underwater vehicles in fragile and confined environments.

### Biological Inspiration

In evolutionary terms there are two possible concepts to respond to mechanical stress: either increase the rigidity of your protective layer, e.g., an exoskeleton or shell, to prevent any deformation, or tolerate certain levels of deformation to minimize the risk of lasting damage (Vincent and Wegst, 2004; Dirks and Taylor, 2012; Wegst et al., 2015).

Especially in unicellular organisms such as ciliates, bacteria or algae, the “tolerate” principle is dominant (King and Beams, 1941; Stocker, 2011; Persat et al., 2015; Sumpio, 2017). These organisms are only separated from their environment by a single membrane or mostly unsclerotised cell walls. Most of these small organisms are also unable to avoid collisions actively; however, due to their small mass and since their “skeleton” is mostly ductile and flexible, they remain unharmed (Persat et al., 2015). The underlying idea of compliance instead of rigidity is applied in soft robotics and modern prosthetics (Trivedi et al., 2008; Belter et al., 2013; Coyle et al., 2018). For underwater vehicles and diving equipment compliance has been used for different actuation strategies (Arienti et al., 2013; Kim et al., 2013; Cianchetti et al., 2015; Laschi et al., 2016), however not yet for structural-skeletal concepts to enhance existing systems. A great advantage of this passively compliant mechanism is its versatility in possible shapes, allowing virtually any system to be equipped with these segmented structures. Here, we show how such a soft and compliant exoskeleton can improve the longevity of an underwater vehicle and reduce the stress, acting upon the system and its environment, in case of a collision.

An additional challenge, which a truly bio-inspired robust system faces, is the ability to compensate for a changing environment, varying carried load, or potential partial system failure. The growing interest in the application of AUVs for exploratory, survey, and economic purposes has led to the development of numerous control strategies, attempting to improve the robustness of such systems to time-variable ocean currents or waves (Elhaki and Shojaei, 2018; Zhong et al., 2018; Shojaei, 2019; Wang et al., 2019; Xia et al., 2019). Recently, the use of neural networks has become increasingly wide-spread in such control applications to account for changing weight distributions of moving manipulators and otherwise unmodeled hydrodynamics (Hernández-Alvarado et al., 2016; Elhaki and Shojaei, 2018; Shojaei, 2019). During the last century, extensive work in the field of biologically inspired adaptive control models has led to a set of principles that enable computation or decision making procedures which are otherwise impractical for traditional mathematical approaches (Lin and Liu, 2010; Hernández-Alvarado et al., 2016; Meena and Devanshu, 2017). Depending on the task of the system, the feedback-based motor controller needs to be adjusted, to work irrespectively of the attached sensory equipment and additional manipulators. To allow a robust, autonomous control of our AUV, we used a genetic algorithm as a means of tuning a system of interlinking PID controllers as a simple three-layered neural network. As the number of weight parameters in our approach is comparably limited, we attempted to use this directed genetic algorithm as a biologically plausible learning strategy, as opposed to backpropagation methods, which are usually employed in more complex network architectures (Riedmiller and Braun, 1993; Hernández-Alvarado et al., 2016). Using a decaying step-size within a genetic algorithm leads to a rapid convergence in its performance over a comparably small number of generations.

To provide a frame of reference for a successfully auto-tuned system, we compared the performance of this approach to the popular heuristic Ziegler-Nichols method for PID tuning (Ho, 1991; Valério and da Costa, 2006). As a proof of concept, we quantified the stabilization performance of the genetic tuning algorithm for the pitch and roll components of the system. Due to the symmetrical shape and weight distribution of our prototype, its behavior is known to be sufficiently predictable to enable an approximated tuning success when using the classic Ziegler-Nichols method (Ho, 1991). In the following sections, we briefly explain the design choices of our newly developed SAUV, outline the implementation and performance of the employed genetic PID tuning algorithm, and quantify the effectiveness of the biologically inspired soft and compliant exoskeleton.

## Materials and Methods

To demonstrate the effect of the compliant exoskeleton principle, derived from unicellular organisms, on a larger scale, a fully functional prototype was built. The resulting ability to absorb kinetic energy was experimentally evaluated using this prototype. To be equally agile in six degrees of freedom, we designed a spherical system with six bidirectional thrusters. This almost holonomic design also eliminates the need for a preferable direction of movement as its drag coefficient remains constant, regardless of the system's orientation (Turton and Levenspiel, 1986).

### The AUV

**Figure 1:**
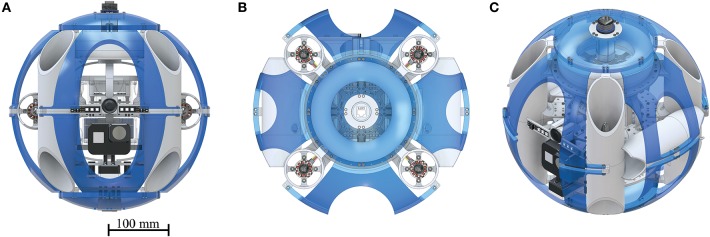
Renderings of constructed SAUV: Soft Autonomous Underwater Vehicle with the compliant exoskeleton (blue, transparent) fitted around it. **(A)** front view, **(B)** top view, **(C)** isometric view. Up to four cameras may be mounted in the lower section of the SAUV. The system is connected to a ground station via a water proof CAT6 connector during the conducted trials to visualize current sensor readings and start the control protocol.

The AUV has a total of six NTM Prop Drive 1000 KV brushless motors (by HobbyKing, Hong Kong) which are each connected to a Turnigy MultiStar 30 ampere electronic speed controller (by HobbyKing, Hong Kong). These ESCs were chosen for their ability to reverse the direction of the brushless motors, using the OneShot125 3D protocol. This principle allows generating thrust in either direction when required, especially in the case of rapid descent or aggressive orientation correction. Four motors are positioned in equal distance to each other and the center of mass, facing upwards. To cancel out the angular momentum, neighboring motors spin in opposite directions, as indicated in the control loop diagram, which is explained in detail in Figure 3. A further two motors are positioned facing forwards on either side of the system. These motors are required to move the system forwards or backwards without adjusting the pitch and to improve upon the system's ability to turn on the spot. CAD renderings of the complete system are shown in Figure 1.

Two ultrasonic distance measuring modules were installed, one in the front, facing forwards, and one in the lower center of the system, facing downwards. These sensors provide the system with readings necessary for primitive obstacle avoidance and, if desired, to keep a constant relative position to the floor. In addition, an altimeter (MS5803 _ 14BA by Sparkfun, Boulder, Colorado, United States) is located in the back of the system to monitor the actual depth relative to the water level by measuring the current water pressure. Two 11.3 V lithium polymer batteries, connected in parallel, are located in a water-sealed container (HPL806 by iSi Deutschland GmbH, Solingen, Germany) in the lower section of the AUV. All other electronic components are contained in a secondary container of the same type in the upper section.

The entire system, at its current stage, is controlled by a Raspberry Pi 3 Model B (by Raspberry Pi Foundation, Cambridge, United Kingdom, Debian release V9.2) which is used to read all sensor input and send motor output commands using i^2^c protocol. For ease of handling, in all experiments conducted underwater, the Raspberry Pi was placed outside the towing tank and connected via a data transmission cable. During the experiments, however, there was no interaction with any of the control components.

### Genetic Algorithm and Preliminary Trials

**Figure 2:**
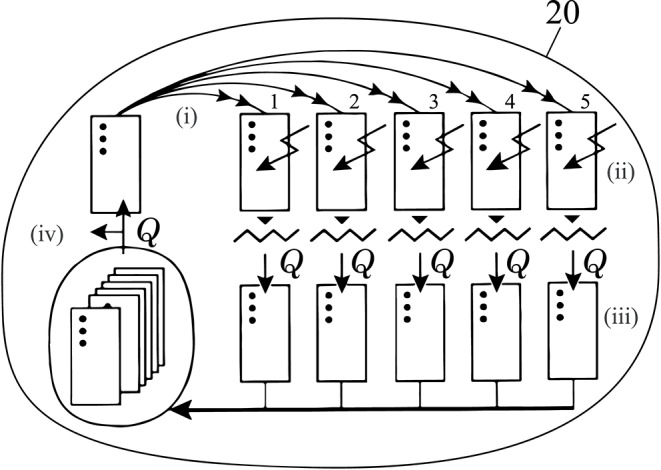
Employed (1, 5)^20^ Evolutionary strategy, depicted using symbols by Rechenberg (Rechenberg, 1989, 2000). **(i)** A single set of variables is duplicated 5 times to produce a new generation. **(ii)** Each offspring is mutated and **(iii)** its fitness is computed based on the *MSE* (Equation 3.2) of the position tracking performance. **(iv)** From this population the offspring with the lowest *MSE* is selected and used as the parent of the next generation. This process is repeated over 20 generations at which point the best performing set of the last generation is determined and the tuning process ends.

The stabilization and position tracking are achieved through a shallow neural network consisting of 4 interlinking PID controllers which were tuned through a simple genetic algorithm following to a (1, 5)^20^ evolution strategy (Rechenberg, 1989, 2000) (Figure 2). These 4 PID controllers are responsible for correcting and stabilizing the systems (1) pitch & roll, (2) yaw, (3) depth, and (4) frontal distance to an obstacle or object of interest. Due to the spherical shape of the system, the weights of the pitch and roll components are shared and thus tuned simultaneously. In general, our tuning approach is executed for one control segment at a time. In this genetic tuning algorithm an “individual,” is represented by a set of corresponding weights for each PID controller within the control loop, and an associated fitness, based on the resulting position tracking performance. The strategy itself can be divided into four distinctive steps: (i) *Reproduction*. Five copies of the fittest offspring of the previous generation are generated. (ii) *Mutation*. Each new offspring is randomly mutated, following Equation 3.1. A randomly generated value −1 ≤ *M* ≤ 1 divided by the number of generations and multiplied by the range specific to the component (*R*_*p*_ = 100, *R*_*i*_ = 10, *R*_*d*_ = 10) is added to the previous weight _*w*_*j*_*gen*−1_ of the set, respectively. (iii) *Evaluation*. After generating these sets, each is applied to the network of controllers and the system, and the fitness of the set is computed over an interval of 5 s in terms of the resulting Mean Squared Error (*MSE*) in the position or angular tracking performance Equation 3.2. The controller error, *e*_*ctrl*_, used in this function corresponds to the currently tuned controller, e.g., *e*_*pitch*_ + *e*_*roll*_, when pitch and roll weights are tuned. (iv) *Selection*. The individual containing the set of values resulting in the lowest *MSE* is selected as the fittest offspring and becomes the parent of the successor generation. The process is then repeated over 20 generations in total. As this selection method is purely based on the system's accelerometer, gyroscope, ultrasonic and altimeter measurements, it does not require manual tracking or user input to execute the tuning process.

(3.1) $$\begin{array}{l} {w_{j}}_{gen}={w_{j}}_{gen-1}+\;\frac{M\cdot R_{j}}{gen} \end{array}$$

(3.2) $$\begin{array}{l} MSE\left(e_{ctrl}\right)=\frac{\sum_{i=0}^{n}{\left(e_{ctrl}\right)}^{2}}{n} \end{array}$$

To demonstrate the effectiveness of the genetic algorithm, a set of preliminary trials were conducted in a custom-built 60 × 60 × 80 cm (width × length × height) aquarium. This setup was chosen to test the algorithm, as in case of unstable behavior, the system could be easily recovered. In front of the aquarium, at a distance of 52 cm between the lens and the front-facing wall of the aquarium, a GoPro Hero 5 Black (by GoPro Inc., San Mateo, United States) on a tripod was positioned in portrait mode (vertically oriented) to record the prototype system within the aquarium (1,920 × 1,080 px at 59,94 fps). To compensate for the lens distortion, all footage was recorded with the integrated field of view set to linear. For later visual tracking of the system within the aquarium, a set of colored 4 × 4 Lego bricks were attached to the top of the horizontally facing tubes o the prototype.

**Figure 3:**
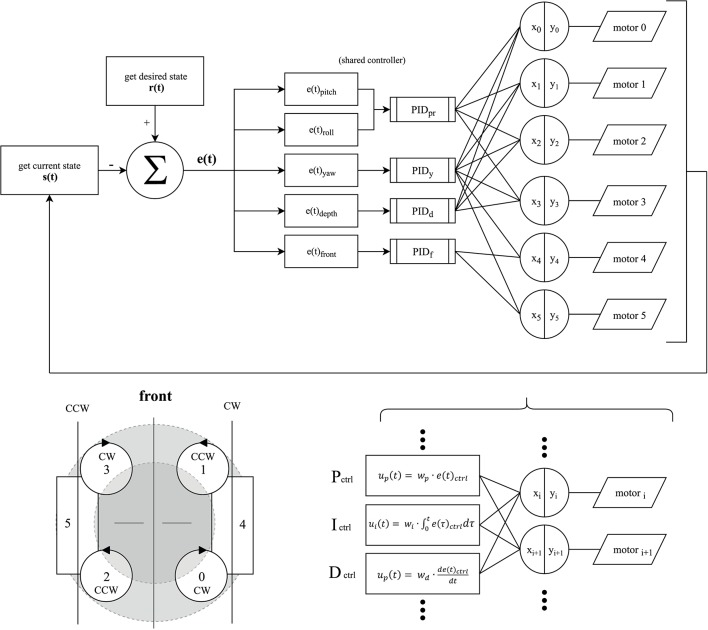
Control diagram of the developed system, consisting of 4 interlinking PID controllers, to allow for accurate position tracking in pitch & roll (*PID*_*pr*_), yaw (*PID*_*y*_), depth (*PID*_*d*_) and distance to an obstacle or object of interest in front of the SAUV (*PID*_*f*_). At the beginning of each increment of the control loop the current state of the system in terms of the vector *s*(*t*) and the desired, or reference, state *r*(*t*) is determined. The reference state can either be the result of direct user input via a gamepad, a pre-programmed route to follow, or a state to maintain. The deviation from the desired state yields the current error of the system *e*(*t*), consisting of the respective values for each controller to correct for. The responses are then mapped to each motor according to the motors' rotation direction (**lower left**) by converting the summed outputs of the PID block *x*_*i*_ into pulse width modulated signals for each ESC denoted as *y*_*i*_. (**lower right**) Each PID controller consists of three components: a proportional *P*_*ctrl*_, an integral *I*_*ctrl*_, and a derivative *D*_*ctrl*_ component. Their respective weights *w*_*p*_, *w*_*i*_, *w*_*d*_ are tuned by a genetic algorithm, depicted in Figure 2.

The system was calibrated outside of the water on a level surface before being lowered into the aquarium. Afterwards, the training program loop was executed, and all sensor data and the fittest PID weights of each generation were saved directly onto the Raspberry Pi. The desired state of the system, in this case, was a constant vertical position, 30 cm above the floor of the aquarium, and a level orientation, without changes in the pitch or roll axis. This mode is referred to as “Hold Position.” After 20 successfully executed generations over the course of 8 min and 20 s, the program was terminated automatically, and the system was lifted out of the water and onto the level surface again. For the next iteration, the system was recalibrated, its batteries recharged and it began with a newly randomized set of input weights. This process was repeated until five iterations had been executed without interference.

For comparison, the heuristic Ziegler-Nichols PID Tuning method (Ziegler and Nichols, 1942) was used as a reference point to which the performance of the genetic algorithm was compared for the pitch and roll components of the controller network. To this end, the respective weights of the pitch and roll nodes were initially set to *w*_*pr*_*d*__ = *w*_*pr*_*i*__ = 0 with the exception of the proportional gain being set to *w*_*pr*_*p*__ = 1. This value was continuously increased until the system reached a stable oscillation, denoted by the constant *K*_*u*_, the ultimate gain. Afterwards, the individual gains for the controller are calculated as described in the original publication (Ziegler and Nichols, 1942) by determining *T*_*u*_, the period of the oscillation at *K*_*u*_ from the recorded video.

(3.3) $$\begin{array}{l} w_{pr_{p}}=0.6\;\cdot \;K_{u} \end{array}$$

(3.4) $$\begin{array}{l} w_{pr_{i}}=\;T_{i}\cdot \;w_{pr_{p}}=\frac{T_{u}}{2}\;\cdot 0.6\;\cdot K_{u} \end{array}$$

(3.5) $$\begin{array}{l} w_{pr_{d}}=T_{d}\cdot \;w_{pr_{p}}=\frac{T_{u}}{8}\;\cdot 0.6\;\cdot K_{u} \end{array}$$

To compare the position-tracking performance of the two tuning methods, the system was recalibrated, lowered into the aquarium, and the “*Hold position*” script is executed. The program loop was then initiated, and after 10 s the recording was started. This time is required, as the system propels itself upwards from the aquarium floor and settles at the desired position. From this point on, the video was recorded over a duration of 10 s, resulting in 599 images for each run. Furthermore, a second condition was tested, in which the system was to ascend and descend rapidly, named “Alter Position.” Under this condition the desired vertical position was switched every 5 s between 30 and 45 cm above the aquarium floor to provoke unstable behavior over a duration of 60 s, resulting in 3,594 images. The performance of all evolutionary iterations after 20 generations and the Ziegler-Nichols method were tested in 5 repetitions in “*Hold Position*” mode. After showing that all iterations converged regarding their resulting position tracking performance (see Figure 6), only iteration one and the Ziegler-Nichols method were then compared in 5 repetitions in “*Alter Position*” mode for the second half of the preliminary trials.

### Exoskeleton

The model of the soft and compliant exoskeleton consists of a total of 10 interchangeable parts, which, when mounted onto the AUV, lead to an overall spherical shape. These parts were manufactured with a dual extruder 3D printer (Dual Extruder Metal Frame BIBO 2, Bibo Automatic Equipment Co., Ltd, Shaoxing, China) using a thermoplastic polyurethane filament (Flexible TPU Transparent, SainSmart Ltd., Lenexa, USA). Additionally, the thruster ducts of the AUV were elongated to provide connecting points for the exoskeleton whereas the elongated sections were created from the same material as the exoskeleton. These elongated sections ensure an equally elastic and compliant behavior of the system regardless of the direction of impact. All other structural components were 3D printed using a ZoneStar Prusa i3 filament printer (ZoneStar Innovation Technology Co., Ltd, Shenzhen, China) with clear PLA filament (by filamentworld, Neu-Ulm).

The radius of the resulting sphere of constant width was chosen to allow at least 30 mm of deformation in any direction before a rigid component would come into contact with its surroundings. The only exceptions to this rule are the mounting points of the laterally placed thruster ducts, in case of a direct 90° impact from either side. As the area, for which this is the case, is negligibly small, compared to the overall size of the system, a smaller circumference of the exoskeleton was favored.

### Evaluation of Exoskeleton Performance

Before conducting velocity and impact trials, the system's motor controller was tuned, using the above described genetic algorithm, with the exoskeleton attached and not retrained for trials without it, as the exoskeleton's effect on the systems total mass is negligible. Furthermore, due to its symmetry, the exoskeleton does not affect the system's center of mass. The total weight of the system, excluding the exoskeleton, is 2,279 g, while the exoskeleton weighs 283 g.

**Figure 4:**
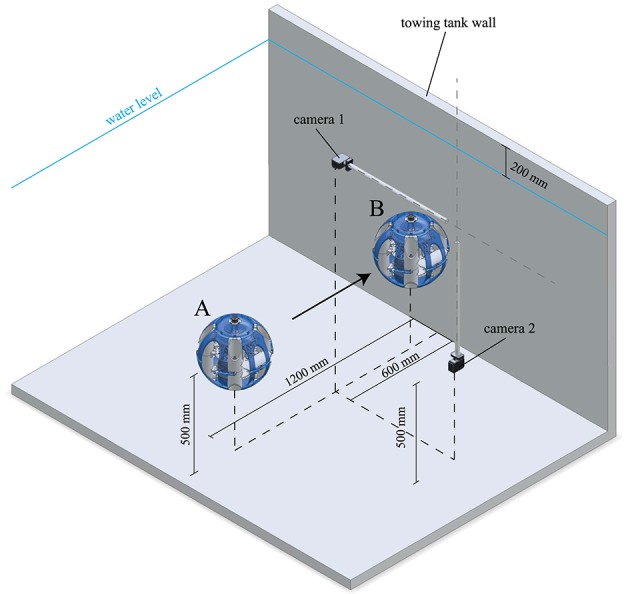
Depicted is a cell shaded rendering of the experimental setup for the conducted “*wet”* impact trials. The SAUV is lowered into the towing tank at position **(A)** and records all IMU data while accelerating toward position **(B)** where it collides with the towing tank wall. After registering the impact, the SAUV stops its motors and backs away from the wall before ascending to the water surface for the following trial. These trials are recorded by two cameras; **camera 1**—GoPro Hero 3+ silver, located 200 mm above the water level, **camera 2**—GoPro Hero 5 black, located underwater at the same depth as the SAUV.

In order to quantify the dampening effect of the soft and compliant exoskeleton, impact trials were conducted in a 1.2 × 2.2 m (depth x width) section of a towing tank. The movement of the AUV was recorded using two synchronized cameras (top, side, 1,920 × 1,080 pixels, 59,94 fps, a shutter speed of 1/120 s, GoPro Hero 5 black and Hero 3+ black, San Mateo, United States). The experimental setup is shown in Figure 4.

The recorded video files were imported into Blender (release 2.79b, GNU General Public License, blender foundation) and analyzed using its internal pattern motion tracking. The resulting trajectories of multiple markers with known distances, to compensate for lens distortion when computing the absolute velocity, were then exported as pixel coordinates using a custom plugin (Blender Motion Tracking Export Plugin, MIT license, www.github.com/BiYonic/blenderMotionExport). An automatic analysis tool for this data was then written in Python 3.6 to compute *v*_max_ and *t*_*v*_max__, as well as to plot the velocity profile of each run.

To determine the maximum velocity, the AUVs motors were set to accelerate to 70% of their maximum velocity to determine *v*_max_ and the required time *t*_*v*_max__ to reach it. As the voltage of the two LiPo batteries affects the maximum velocity, they were fully recharged in between impact trials with and without the attached exoskeleton. At the beginning of each trial, the AUV was lowered into the towing tank at a distance of approximately 1.2 m from a wall of the tank, facing it directly. The AUV automatically positioned itself at a depth of 0.5 m below the water level and corrected its drift and orientation steadily for 5 s, settling at a steady position before beginning to accelerate toward the wall in front of it. The AUV then set its horizontally oriented thrusters to 70% of their maximum output to allow for yaw corrections while driving forwards and began logging the acceleration and angular velocity of the system from its inertial measurement unit (IMU) at a refresh rate of 59 Hz ± 1.3. The thrust was kept constant until an impact was registered by the IMU which prompted the horizontal motors to stop and then reverse their direction to move away from the wall. After moving backwards for 1 s, the system was set to hold its current position for 5 s before stopping the recording of IMU data and ascending to the water surface to be redeployed for the next trial.

To derive the true net acceleration and to compute the resulting velocity from the recorded IMU data, gravity and orientation compensation was performed according to Nistler and Selekwa (2011) and implemented in python 3.6. This way the effects of the tilt in the pitch, roll, and yaw axes on the acceleration in cartesian space were accounted for and the position relative to the starting point of the system, as well as its velocity, could be computed accurately. Both inertial and gyroscope measurements are required for this correction. The recorded duration of a single trial does not exceed 20 s; thus, no additional filters were applied to compensate for possible sensor drift, as it is negligible. The compared magnitude of the net acceleration, as shown in Equation 3.7 is dependent on the following components: ${\ddot{\vec{r}}}_{xyz}$, the corrected acceleration vector, ${\ddot{\vec{r}}}_{XYZ}$, the input acceleration vector including gravity, as recorded by the inertial measurement unit, ${\overset{∙}{\vec{r}}}_{XYZ}$, the computed velocity vector, ${\vec{r}}_{XYZ}$, the computed relative position vector, and *J*, the *X*- *Y*- *Z* rotation matrix, in Equation 3.6. For further details, refer to the original publication by (Nistler and Selekwa, 2011).

(3.6) $$\begin{array}{l} {\ddot{\vec{r}}}_{xyz}=J{\ddot{\vec{r}}}_{XYZ}+2\overset{∙}{J}{\overset{∙}{\vec{r}}}_{XYZ}+\ddot{J}{\vec{r}}_{XYZ} \end{array}$$

(3.7) $$\begin{array}{l} |{\ddot{\vec{r}}}_{xyz}|=\;\sqrt{{\ddot{\vec{r}}}_{x}^{2}+{\ddot{\vec{r}}}_{y}^{2}+{\ddot{\vec{r}}}_{z}^{2}} \end{array}$$

**Figure 5:**
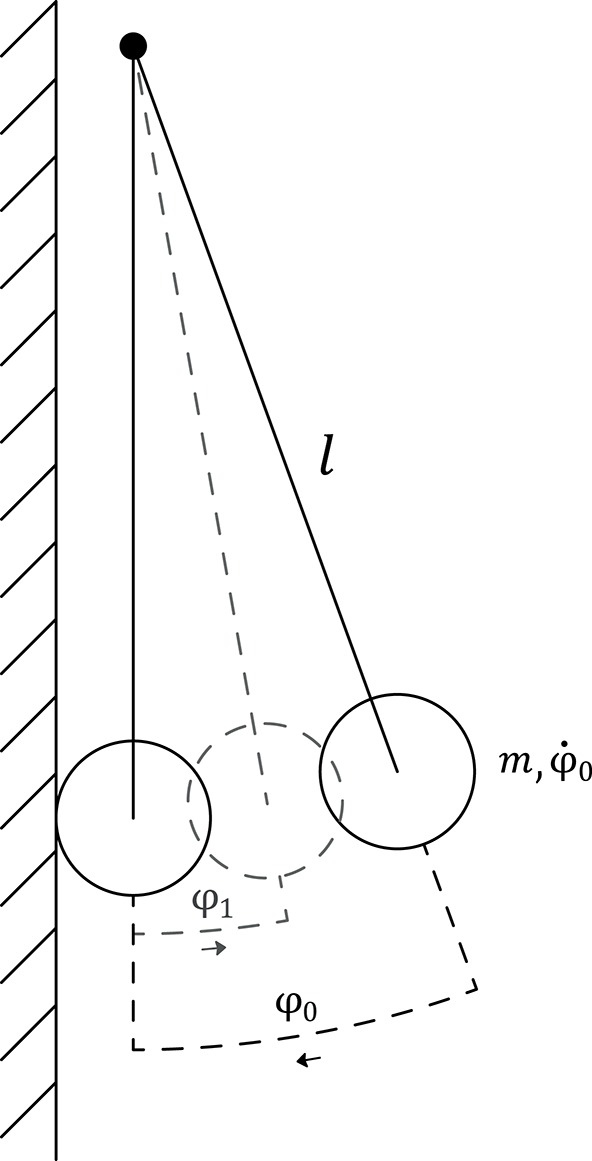
“*dry”* Experimental setup of the gravity pendulum. The SAUV is assumed to be a point mass *m*, initially at rest prior to release $\left({\overset{∙}{\varphi }}_{0}=0\right)$ at an angle of $\varphi_{0}=15^{∙}$ ±0.2°. The maximum angle φ_1_ after the impact with a rigid wall is measured to compute the coefficient of restitution of *C*_*R*_.

To quantify the effect of the soft and compliant exoskeleton itself, also “dry” impact trials were conducted with the SAUV as a simple gravity pendulum and a rigid wall, depicted in Figure 5. The initial value problem-setup was taken with a length of the pendulum of *l* = 75 *cm*, the SAUV itself was assumed to be a point mass and the initial conditions φ_0_ = 15 ± 0.2 and ${\overset{∙}{\varphi }}_{0}=0.$ The impact trials were conducted with and without the attached exoskeleton parts with *n* = 5, respectively, to record the resulting maximum angle after a collision, denoted as φ_1_. The true net acceleration was also plotted according to Equations (3.6) and (3.7) with 20 repetitions in each condition.

### Statistical Evaluation

During the described preliminary trials, the Shapiro-Wilk test was used to test for normal distribution. Whenever more than two samples were tested for homogeneity of variances, the Levene's test was performed. Otherwise, a two-sample *F*-Test was used. For multi-sample comparison of the mean values, the Kruskal–Wallis rank-sum test was used. If significance occurred either the Dunn's test or the Wilcoxon rank-sum test was used *post-hoc* to identify the responsible subgroups. Whenever normally distributed samples with homogeneity of variances were found between two samples, the Welch Two Sample *t*-test was used to test their mean.

To compare the computed net acceleration and velocities in trials with and without the mounted soft and compliant exoskeleton, the following cascade of tests was performed; The Shapiro–Wilk test was used to test for normal distribution in each data set, followed by the Barnett test to test for homogeneity of variances. Since in all tests, both criteria were met, a two-sample student *t*-test was performed to compare the mean between the two samples. For every velocity and impact test, the sample size was *n* ≥ 10 with a significance level of α = 5%. All statistical evaluations were performed in python 3.6, using the library *scipy.stats*.

## Results

### Genetic Algorithm Performance in Preliminary Trials

**Figure 6:**
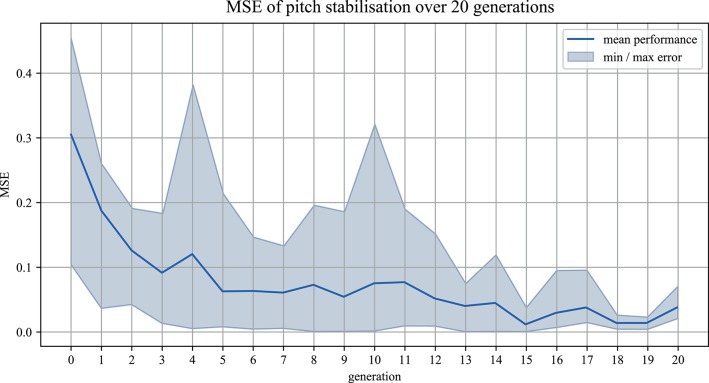
The *MSE*(*e*_*pitch*_) of the best performing parameter sets in the pitch component over 20 generations is shown, recorded during the genetic algorithm tuning process. The light blue area indicates the range of resulting position tracking performances over the 5 repeated iterations and the dark blue line the mean performance in each generation.

**Figure 7:**
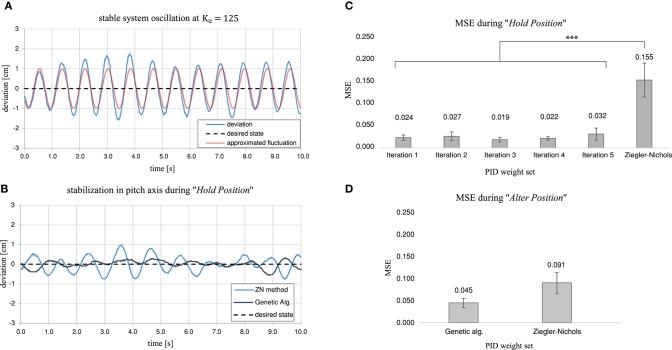
Summarizing the findings of the preliminary trials to evaluate the effectiveness of the genetic algorithm for tuning the nodes related to pitch control. **(A)** The deviation between the front and rear tracking points in centimeters, located on the rims of the horizontal thruster ducts. The stable oscillation achieved during the tuning process of the Ziegler-Nichols algorithm can be approximated by a sinusoidal wave with a period of *Tu* = 0.825. **(B)** Example stabilization in pitch axis over 10 s in “*Hold Position*” mode for the Ziegler-Nichols Tuned nodes compared to the performance of the genetic algorithm after 20 generations. **(C)** Resulting Mean Square Error in stabilization performance in “*Hold Position*” mode for all 5 iterations after 20 generations each compared to the performance of the Ziegler-Nichols method. **(D)** Resulting Mean Square Error in stabilization performance in “*Alter Position*” mode, during rapid vertical position changes, for the genetic algorithm from iteration 1 after 20 generations compared to the performance of the Ziegler-Nichols method.

Over the course of 20 generations in 5 separate iterations, a total number of 500 individual value sets have been applied and tested in “*Hold Position*” mode during the genetic algorithm tuning phase. The fitness of each individual was determined by its resulting MSE, computed during the runtime of the tuning phase by directly comparing the filtered sensor deviation of the inertial measurement unit within each generation. By comparing the MSE after 20 generations of all iterations in “*Hold Position*” mode to one another, convergence could be shown for the performance of the genetic algorithm tuning (Kruskal–Wallis = 0.81231, *p* = 0.9368), (Figures 6, 7).

During our preliminary trials, for comparison, the system was tuned using the classic Ziegler-Nichols method. Steady oscillation of the system was achieved when the proportional gain of the pitch and roll segment *w*_*pr*_*p*__ reached *Ku* = 125. The oscillation period at that point was determined by an analysis of the footage obtained executing “Hold Position.” *Tu* is therefore equal to 0.825. The approximated deviation (*t*), in terms of the vertical distance between the rearand front tracking points of the system, can be described by $d\left(t\right)=\sin\left(\frac{2\pi \;\cdot t}{T_{u}}\right)$ and a comparison to the recorded video data is shown in Figure 7A. After calculating *w*_*pr*_*p*__, *w*_*pr*_*i*__, and *w*_*pr*_*d*__, the stabilization performance in “*Hold Position*” mode led to an MSE of 0.15463 ± 0.0772. An example of this stabilization method and the performance achieved through the genetic algorithm are shown in Figure 7B.

The performance achieved with the use of the genetic algorithm and the Ziegler–Nichols method was significantly different during “*Hold Position*” (Kruskal Wallis = 12.308, *p* = 0.00903, with all Dunn *p* < 0.05). While the MSE over all iterations after 20 generations was between 0.0188 ± 0.0108 for iteration 3, and 0.0316 ± 0.0225 for iteration 5, the MSE for the Ziegler-Nichols Method was 0.15463 ± 0.0772. The MSE for all compared performances, as well as the maximum absolute error *e*_*M*_ are shown in Figure 7C and Table 1.

**Table 1:** Experimental results of the pitch and roll controller tuning, quantifying the tracking error *e*_*ctrl*_ of the system in hold position and alter position mode.

**Mode**	**Hold position**		**Alter position**	
	**GA**	**ZN**	**GA**	**ZN**
MSE *e*_*M*_	0.025	0.155	0.045	0.091
	0.575	0.971	0.741	0.977

*The MSE (Mean Squared Error) and e_M_ (maximum absolute error) are shown. **GA** (Genetic Algorithm) and **ZN** (Ziegler-Nichols) refer to the employed tuning method*.

Our experiments in “*Alter Position*” mode, with rapid vertical position changes, however, showed no significant difference in the MSE of the pitch and roll stabilization when comparing the genetic tuning method to the Ziegler-Nichols method (Kruskal-Wallis = 3.1527, *p* = 0.0758). A comparison of the MSE is shown in Figure 7D. There was also no significant difference between the MSE of iteration 1 in “*Hold Position*” mode and “*Alter Position*” mode (Welch Two Sample *t*-test = −2.3097, *p* = 0.0537), or the MSE of the Ziegler-Nichols Method between “*Hold Position*” mode and “*Alter Position*” mode (Welch Two Sample *t*-test = 1.5641, *p* = 0.1685).

### Velocity Profiles

**Figure 8:**
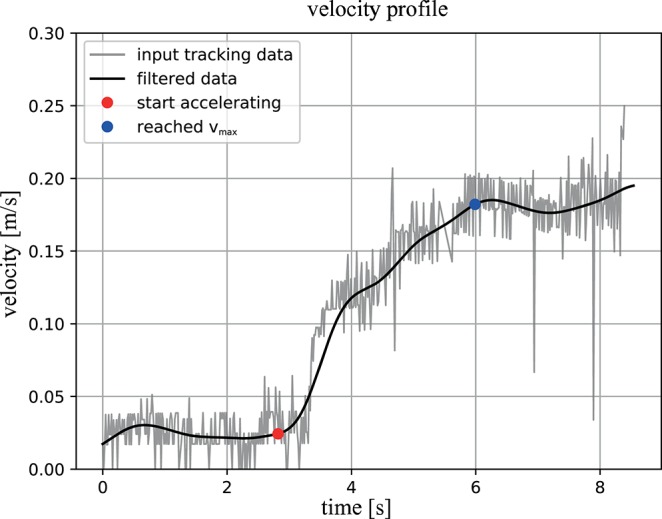
Example velocity profile: the measured absolute velocity of the system is indicated by the gray graph and for the black graph the same data was used, yet a Butterworth frequency filter was applied to account for the noise in the tracking data. The red dot indicates the point in time when the ROV began accelerating and the blue dot marks the instant *v*_max_ was reached. The interval between these points is equivalent to Δ*t*_*v*_max__.

Over a total of 11 initial trials *v*_max_ was measured to be 0.168 ± 0.072 *m*/*s* and the required time to accelerate to that velocity *t*_*v*_max__ was 3.6 ± 0.68 *s*. A representative velocity profile is depicted in Figure 8. These results show that when requiring the velocity of the system to be equal to *v*_max_ prior to the impact, the distance to the wall must be ≥ 0.35 *m*. As the distance to the wall within the impact trials is ≈ 1.2 *m* the AUV was able to reach the desired velocity in every trial.

**Figure 9:**
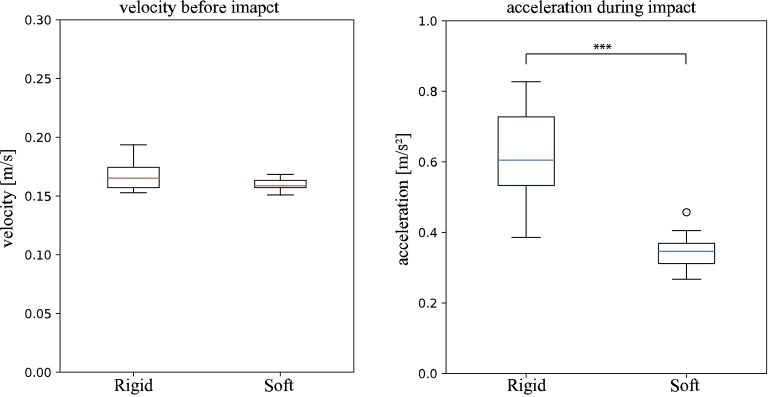
“Wet” Impact trials (conducted underwater in a towing tank). velocity *v*_max_ (**Left**): The boxplots show the computed velocity in *m*/*s* of the system 2 s prior to the registered impact. **Rigid** indicates the data produced by ROV without the mounted soft exoskeleton and **Soft** the data produced with the soft exoskeleton. The computed velocities are not significantly different, indicating there is no effect of the soft exoskeleton on the maximum velocity. Peak net acceleration *a*_max_ (**Right**): The peak acceleration *a*_max_ measured during all impact trials with (**Soft**) and without (**Rigid**) the mounted soft exoskeleton. A significant difference between the setup with and without the mounted soft exoskeleton is indicated by *** *p* returned from a two sample students *t*-test.

The computed average velocity from the IMU data for both setups, with and without the mounted exoskeleton, 2 s before the registered impact indicates that this is the case. Figure 9 shows the velocities measured during this time. The velocity 2 s prior to the impact without the exoskeleton was 0.167 ± 0.012 *m*/*s* and with the exoskeleton 0.160 ± 0.0049 *m*/*s*. Both samples are normally distributed and have homogeneous variances. The student *t*-test returned a *p*-value of 0.118, indicating no significant difference in the mean velocity with and without the mounted exoskeleton.

### Impact Trials

**Figure 10:**
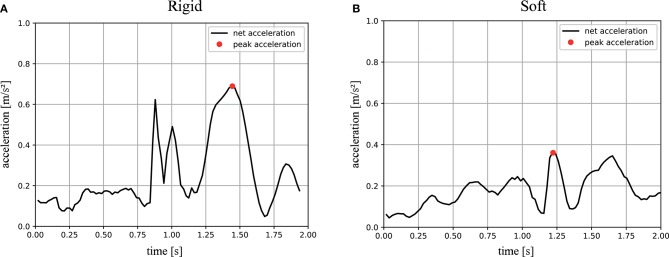
Magnitude of the net acceleration during underwater trials: the black graphs show the magnitude of the net acceleration, corrected for orientation and gravity, of a representative trial, excluding gravity, in *m*/*s* during an impact trial without the soft exoskeleton **(A)** and with the soft exoskeleton **(B)**. The data is plotted for 1 s before and after the impact, meaning the onset of the impact was registered by the system at 1 s on the x-axes. The red dots indicate the peak acceleration measured during this time. The constant non-zero magnitude is caused by motor corrections and minor sensor noise.

In total, 11 “wet” trials with and without the soft exoskeleton were evaluated. Representative acceleration profiles of the “wet” trials are shown in Figure 10. These plots were generated to show the magnitude of the net acceleration, according to Equation 3.7, 1 s before and after the registered impact, over a total span of 2 s. The recorded peak net acceleration in the trials without the soft exoskeleton was 0.616 ± 0.141 *m*/*s*^2^ and in the trials with the soft exoskeleton 0.347 ± 0.05 *m*/*s*^2^ as shown in Figure 9. Again, both samples are normally distributed and have homogeneous variances. A students *t*-test shows a significantly lower impact acceleration for the trials with the exoskeleton (*p* < 0.001).

**Figure 11:**
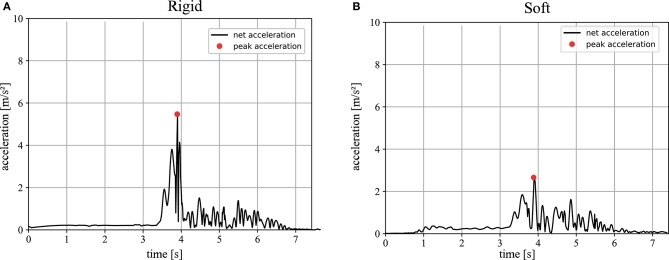
Magnitude of the net acceleration during pendulum trials: the black graphs show the magnitude of the net acceleration, corrected for orientation and gravity, of a representative trial, excluding gravity, in *m*/*s* during an impact trial without the soft exoskeleton (**A**) and with the soft exoskeleton (**B**). The data is plotted for the entire duration of the trial, with the release of the system occurring after 3 s and recording until it comes to a rest. The red dots indicate the measured peak acceleration.

**Figure 12:**
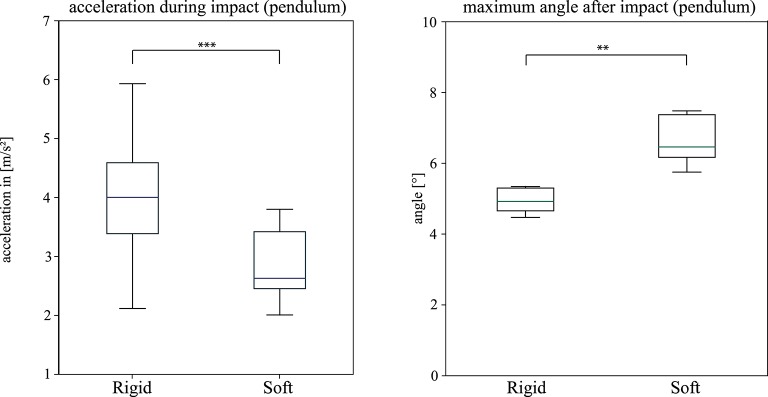
“Dry” Impact trials (gravity pendulum). Comparison Boxplot Peak net acceleration (**Left**): The peak acceleration *a*_max_ measured during all impact trials with (**Soft**) and without (**Rigid**) the mounted soft exoskeleton. _φ_1_max_ (**Right**): The boxplots show the measured maximum assumed angle of the pendulum after the impact of the system. A significant difference between the setup with and without the mounted soft exoskeleton is indicated by ** for a *p*-value < 0.01 and *** for a *p*-value < 0.001 returned from a two sample students *t*-test.

The “dry” trials deliver a maximum angle after a collision of φ_1_ = 6.63 ± 0.75 for the soft trials and φ_1_ = 4.93 ± 0.38 for the rigid ones, which lead to a coefficient of restitution of *C*_*R*_ = 0.432 ± 0.06 for the soft and *C*_*R*_ = 0.329 ± 0.02 for the rigid trials. Representative acceleration profiles are shown in Figure 11. The recorded peak net acceleration in the “dry” trials without the soft exoskeleton was 4.1 ± 1.09 *m*/*s*^2^ and in the trials with the soft exoskeleton 2.87 ± 0.56 *m*/*s*^2^ as shown in Figure 12. Because the soft exoskeleton does not substantially affect the mass of the AUV, the acceleration is proportional to the acting force, leading to a factor of nearly 1.5.

## Discussion

Inspired by the passive and compliant biological concept, we here present a soft and compliant exoskeleton, fitted to our newly developed SAUV (Soft Autonomous Underwater Vehicle). The SAUVs position tracking is accomplished by a shallow artificial neural network consisting of interlinking PID controllers which are tuned through a genetic algorithm.

### Performance of the Genetic Algorithm to Control Motor Outputs

Our results show that using our SAUV as a training platform, application of the Ziegler–Nichols method resulted in an insufficient stabilization compared to the performance of the genetic algorithm. The Ziegler-Nichols PID tuning method is widely used in the controlling of plants, and over the course of the second half of the twentieth century various adjusted versions of it have been published for specific use cases (Ho, 1991; Valério and da Costa, 2006). The classic method used in this research has shown that its aggressive correction due to its high proportional gain leads to an overcompensation which is in itself not consistent yet yields an overall higher Mean Squared Error (MSE).

Other versions of this tuning method take this form of overshoot into account and provide a more appropriate response which can be taken into consideration if revisited in a future study (Ho, 1991). Nevertheless, the fact that such manual tuning methods take a considerable amount of time to perform with uncertain results makes it overall impractical for further use in this scenario. The process of determining *Ku* at a stable oscillation with adequately small increments as well as the evaluation required to compute *T*_*u*_ took a total of 6 h. Applying the genetic algorithm, following a (1, 5)^20^ evolutionary strategy provided significantly better results within only 8:20 min. Even if a reliable outcome, on par with the genetic algorithm method, could be expected from a heuristically weighted method, the sheer amount of time going into the tuning process would not justify its use. Especially when considering that this method is only applicable in an offline scenario compared to recently published online applications for PID tuning using neural networks (Hernández-Alvarado et al., 2016).

As the learning process itself was performed exclusively during “Hold Position” it is apprehensible that the performance of this method was less accurate under rapidly changing conditions in “*Alter Position*” mode, which aims at producing large disturbances. To a certain extent, this behavior can be interpreted as overfitting of the tuning parameters. The purposely chosen small size of the aquarium might also be the limiting factor at this point, as after a short period of time the creation of vortices within the water could be observed when the system was at work. Because the duration of a single repetition for “*Alter Position*” mode was six times longer than the duration of a “Hold Position” mode repetition, it would have to be further investigated whether a decrease in performance could be observed over time which would hint at a negative influence of the testing conditions. It is important to note that the converging performance was not necessarily due to similar combinations of weights but rather multiple local minima of the MSE which all led to acceptable behavior. The simple genetic algorithm, employing a “winner-takes-all” approach paired with fitness function determined by a single dimension of performance, likely needs to include further parameters, training generations, or greater variability in the training conditions to approach a more globally optimal behavior (Lin and Liu, 2010; Jayachitra and Vinodha, 2014; Meena and Devanshu, 2017).

In the future, to improve upon the system's position tracking performance under more complex conditions, control strategies explored in recent publications are to be considered. Especially the current lack of a feed-forward model to control the SAUV, rather than a purely feedback-based control approach needs to be addressed (Elhaki and Shojaei, 2018; Shojaei, 2019; Wang et al., 2019) as well the potential issue of actuator saturation by utilizing anti-windup compensators (Galeani et al., 2009; Cui et al., 2016; Xia et al., 2019).

In general, the preliminary trials have confirmed the suitability of a biologically plausible genetic algorithm to automatically tune specific sections or the entirety of the controller architecture of such an underwater vehicle. The shallow network structure consisting of interlinking PID controllers can be effectively tuned after comparably few generations and performs significantly better than the heuristic Ziegler-Nichols tuned controller. Major advantages of this implementation include (a) the ability to retrain the system quickly when additional equipment is attached, (b) changing the fitness parameter to include, e.g., the power consumption of the system to select for energetically efficient control strategies, and (c) using the previously adapted parameters to fine-tune the system's response during use. All of the aforementioned advantages are to be explored in-depth as the development of the SAUV continues.

### Performance of the Soft and Compliant Exoskeleton

The impact trials in this study clearly show that soft exoskeletons are a viable means to increase the safety and longevity of underwater vehicles while preserving their performance and without limiting the use of sensors or the attached cameras' fields of view. The conducted experiments show that the biologically inspired soft and compliant exoskeleton significantly reduces the net acceleration during an impact without restricting the AUVs performance in terms of its maximum velocity. A lower acceleration indicates lower mechanical stress on the system itself as well as lower reaction forces on its environment. Secondary peaks in the computed net acceleration, as in Figure 9, appear whenever there are multiple contact points with the wall during an impact. These peaks are less dominant when the soft exoskeleton is attached as its spherical shape leads to a smaller number of contact points but a larger overall contact area. The higher coefficient of restitution of *C*_*R*_ = 0.432 ± 0.06 for the soft and compliant exoskeleton further indicates a greater reversible deformation. The “dry” trials indicate a reduction in the resulting impact forces of at least 30% when the exoskeleton is mounted on the AUV. Furthermore, in the “dry” trials an increase in the duration of the impact, indicated by the stretched acceleration curves, allows more time for the system to deform. The most striking result lies in the “wet” trials, where a mean reduction of 56% in the occurring peak accelerations can be demonstrated.

To further analyse the effects of an impact on such a system, conducting numerical simulations is a fitting approach. Precise computation of the resulting forces and stresses on the system cannot be performed from IMU data alone, as parameters such as stiffness, damping ratios, as well as contact area vary with the direction and point of impact (Tempelman et al., 2012). Nevertheless, the peak magnitude of the net acceleration is an adequate measure of the severity of an impact.

There is a broad spectrum of applications for this soft exoskeleton. Especially since its use enables divers to safely interact with almost any existing ROV and AUV of any scale as they would no longer have to fear the consequences of an impact by the system. An AUV fitted with a soft exoskeleton may, for example, accompany divers and provide them with lighting of their surroundings or objects in focus to ensure the diver is able to use both of their hands while operating. It is also feasible to send an AUV alongside divers until they reach a machine, crevice or archaeological site which is not directly accessible to the divers. A small AUV with a soft exoskeleton can enter these areas to collect data or perform other tasks. Using our constructed SAUV in a flooded mine, we aim to use it for an automated gathering of omnidirectional video footage for photogrammetry and ultimately an automated 3D reconstruction of previously unmapped or inaccessible cave systems.

Another possible application for such an exoskeleton is the enhancement of scooters and other diving equipment used to propel divers or keep them at a certain depth. Especially when more than one person is operating underwater at the same time, which is a key safety requirement (Buzzacott et al., 2009), the risk of a collision or interference with other machinery must be kept to a minimum. The same principles of the soft exoskeleton may be applied here which make the overall use of these systems safer. In conclusion, the application of our proposed soft exoskeletons results in the following benefits to any underwater vehicle or appliance:

- greatly improves the ability of ROVs and AUVs to operate in an unpredictable environment
- allows for safe operation in close contact with human divers
- reduces the risk of damage to the ROV or AUV
- reduces the risk of damage to the fragile underwater environment
- is a cost-effective means to reduce the need for perfect obstacle avoidance
- is a cost-effective means to upgrade the operational scope and safety of existing systems.

## Data Availability Statement

The datasets generated for this study are available on request to the corresponding author.

## Author Contributions

FP built the AUV, programmed the ANN and collected the experimental data. All authors analyzed the data, interpreted the results, contributed equally to the manuscript and approved the submission.

### Conflict of Interest

The authors declare that the research was conducted in the absence of any commercial or financial relationships that could be construed as a potential conflict of interest.

## Supplementary Material

Supplementary Video 1 (SAUV_Demo.mp4). A short introduction and demonstration of the developed prototype. Footage adapted from Mantismundi (2016) and Smith (2012). Music by Chris Zabriskie (CC BY 4.0).

Supplementary Video 2 (Comp_Rigid_compressed.mp4). Example recording (top and side view) during an impact trial without the soft and compliant exoskeleton attached.

Supplementary Video 3 (Comp_Soft_compressed.mp4). Example recording (top and side view) during an impact trial with the soft and compliant exoskeleton attached.
